# Intermedin protects against myocardial ischemia-reperfusion injury in diabetic rats

**DOI:** 10.1186/1475-2840-12-91

**Published:** 2013-06-18

**Authors:** Hong Li, Yunfei Bian, Nana Zhang, Jia Guo, Cheng Wang, Wayne Bond Lau, Chuanshi Xiao

**Affiliations:** 1 Department of Cardiology, Shanxi Medical University, 030001 Taiyuan, Shanxi, China; 2 Department of Emergency Medicine, Thomas Jefferson University, Philadelphia, PA 19107 USA

**Keywords:** Intermedin, Ischemia-reperfusion, Diabetes, Oxidative stress, Apoptosis, Inflammatory

## Abstract

**Background:**

Diabetic patients, through incompletely understood mechanisms, endure exacerbated ischemic heart injury compared to non-diabetic patients. Intermedin (IMD) is a novel calcitonin gene-related peptide (CGRP) superfamily member with established cardiovascular protective effects. However, whether IMD protects against diabetic myocardial ischemia/reperfusion (MI/R) injury is unknown.

**Methods:**

Diabetes was induced by streptozotocin in Sprague–Dawley rats. Animals were subjected to MI via left circumflex artery ligation for 30 minutes followed by 2 hours R. IMD was administered formally 10 minutes before R. Outcome measures included left ventricular function, oxidative stress, cellular death, infarct size, and inflammation.

**Results:**

IMD levels were significantly decreased in diabetic rats compared to control animals. After MI/R, diabetic rats manifested elevated intermedin levels, both in plasma (64.95 ± 4.84 pmol/L, p < 0.05) and myocardial tissue (9.8 ± 0.60 pmol/L, p < 0.01) compared to pre-MI control values (43.62 ± 3.47 pmol/L and 4.4 ± 0.41). IMD administration to diabetic rats subjected to MI/R decreased oxidative stress product generation, apoptosis, infarct size, and inflammatory cytokine release (p < 0.05 or p < 0.01).

**Conclusions:**

By reducing oxidative stress, inflammation, and apoptosis, IMD may represent a promising novel therapeutic target mitigating diabetic ischemic heart injury.

## Background

Large epidemiological studies have demonstrated acute coronary syndrome (ACS) is 2–3 times more prevalent in diabetics than the general population [1,2]. Females are at greater risk for acute myocardial infarction (MI) compared to male diabetic patients [3]. Diabetic patients are more susceptible to myocardial ischemia/reperfusion (MI/R) injury than non-diabetics, with greater mortality and resultant heart failure [4-6]. Diabetes is a comorbidity of 50% of MI mortalities [7,8].

Select pharmacologic agents reducing myocardial injury in non-diabetic animal models are ineffective in diabetic animal models [9]. Intermedin (IMD), also known as adrenomedullin 2 (ADM2), belongs to the calcitonin gene-related peptide (CGRP) superfamily. Peptide fragments (IMD1–47, IMD8–47, and IMD1–53) are generated from pre-proIMD by proteolytic cleavage [10]. Among the three degraded fragments, IMD1–53 exhibits the most potent biological cardiovascular effect [11]. IMD has been shown to have pathophysiological effect in multiple disease processes involving the circulatory and renal systems [12] and congestive heart failure [13]. IMD augments cardiac contractility [14], inhibits collagen synthesis, attenuates proliferation of cardiac fibroblasts [15], and attenuates cardiomyocyte hypertrophy [16].

Recently, intermedin has been demonstrated to protect human macrovascular, microvascular, and cardiac non-vascular cells against I/R injury via AM(1)-receptor signaling [12]. Furthermore, IMD1–53 exerts potent cardioprotective effects against acute rat ischemic injury [17], inhibiting endoplasmic reticulum stress via PI3 kinase-Akt signaling [18], and activating cardioprotective Akt/GSK-3beta signaling, decreasing mitochondrial-mediated myocardial apoptosis [19]. Hyperglycemia downregulates the cardioprotective peptide adrenomedullin in streptozotocin-induced diabetic rats, potentially exacerbating diabetic cardiomyopathy and left ventricular dysfunction [20]. However, whether IMD1-53 has any protective effect in the diabetic condition is completely unknown.

As diabetic ischemic heart disease is a prevalent clinical problem with significant morbidity and mortality, IMD1-53 may have promising therapeutic potential. Therefore, the aims of the current study were 1) to determine whether IMD1–53 may protect diabetic hearts against MI/R injury, and if so, 2) to determine the underlying responsible mechanisms.

## Methods

### Animals and groups

Male Sprague–Dawley rats (250-300 g, Shanxi Medical Laboratory Animal Center, China) were housed with free access to standard rat chow and water in accordance with the principles of the Animal Management Rule of the Ministry of Health, People’s Republic of China (Document No. 55, 2001) and the Guide for the Care and Use of Laboratory Animals published by the US National Institutes of Health (NIH Publication No. 85–23, revised, 1996). All study protocols were approved by the Shanxi Medical University Animal Care Committee (Shanxi, China). Rats were randomly assigned to five different groups: non-diabetic sham (NS, n = 12), non-diabetic + ischemia/reperfusion (NIR, n = 12), diabetic sham (DS, n = 15), diabetic + ischemia/reperfusion (DIR, n = 15), and diabetic + ischemia/reperfusion + IMD treatment (IMD, n = 15). In the IMD treatment group, IMD 1–53 (dose 20 nmol/kg, Phoenix Pharmaceutical, Inc. Belmont, henceforth referred to as IMD) [17] was infused 20 minutes after MI onset, via the left femoral vein over a period of 10 minutes.

### Diabetes induction

**Figure 1:**
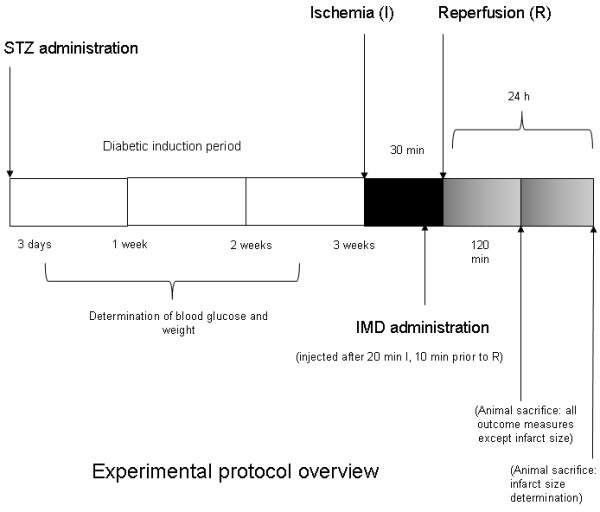
**Schematic representation of experimental design.** Diagram demonstrates the diabetic induction period, ischemia/reperfusion period, IMD administration time point, and animal sacrifice end points.

Diabetes was induced by intravenous injection STZ (Sigma Chemical Co). STZ was dissolved in citrate buffer (pH 4.5), and administered in a single 55 intraperitoneal (IP) mg/kg injection [21]. Rats were fasted overnight before STZ injection. Control rats were injected with buffer only (10 mM citrate buffer, pH 4.5) after an identical fasting period. Female hormonal profile resistance to STZ-induced diabetic phenotype without testosterone supplementation is a documented phenomenon [22] Therefore, in consistent fashion with multiple other investigations employing a similar diabetic model, only male Sprague–Dawley rats were utilized in the current study (Figure 1). Tail blood glucose samples were obtained from each rat after 3 days, 1, 2, and 3 weeks after STZ administration via glucometer (Glucotrend, Roche). Rats exhibiting hyperglycemia (fasting blood glucose ≥16.7 mmol/L, from at least three samplings) were considered to have diabetes. The mortality rate of rats exposed to STZ treatment was 26.7% (12 of 45 total rats subjected to STZ treatment died).

### Myocardial ischemia and reperfusion model

3 weeks after the initial STZ or control injection, SD rats were anesthetized by IP injection of 7% chloral hydrate (350 mg/kg). A left thoracotomy and pericardiotomy were performed. The left coronary artery was dissected above the first diagonal branch and ligated immediately proximal to the left circumflex arterial origin with silk thread. Slipknot-induced occlusion commenced for 30 minutes. R wave amplification and ST segment depression were observed immediately in lead II of the attached electrocardiogram. Myocardium distal of the ligation line darkened, indicating myocardial ischemia (MI). After 30 minutes MI, the slipknot was released for 120 minutes, allowing reperfusion (R) [23]. Blood was collected after R via cardiac puncture and centrifuged at 2000 × g for 10 minutes. Serum and plasma were stored at −80°C for further analysis. Rats were sacrificed via direct intraventricular 2.56 M KCl injection [24]. Hearts were removed and rinsed with ice-cold phosphate buffered saline. Ventricular tissue was immediately frozen in liquid nitrogen and stored at −80°C.

### Hemodynamic measurements

Changes in left ventricular developed pressure (LVDP) and the maximal rates of increase and decrease in LV pressure (±dp/dtmax) were monitored by a Mikro-Tip® Catheter Pressure Transducer (BL420F-Powerlab, Taimeng Technology Co., Ltd.), inserted into the left ventricular cavity via the right common carotid artery. Data was continuously recorded at the onset of reperfusion.

### Radioimmunoassay for plasma IMD levels

Blood samples were anticoagulated with Na_2_EDTA, 1 mg/mL aprotinin, and 500 K IU heparin. Plasma was separated by centrifugation (1600 × *g* for 15 minutes at 4°C) and stored at −80°C [25]. Plasma was loaded onto a Sep-Pak C18 cartridge (Phoenix Pharmaceutical) and pre-equilibrated with 0.5 mmol L-acetic acid, and the adsorbed material was eluted with 4 ml 50% CH_3_CN containing 0.1% trifluoroacetic acid. After lyophilization, the residue was dissolved in radioimmunoassay buffer, and analyzed per manufacturer protocol. IMD radioimmunoassay kits (Phoenix Pharmaceutical, Inc.).

### Serum biochemical analysis

Serum LDH, CK-MB, tumor necrosis factor alpha (TNF-α), interleukin 6 (IL-6), and interleukin 1-beta (IL-1β) levels were determined via rat ELISA kit (Nanjing Jiancheng Bioengineering).

### Cardiac tissue MDA, SOD, NOS, and NO measurement

Cardiac MDA, SOD, NOS, and NO levels were determined to assess oxidative stress as described previously [26-28]. After the 2 hour reperfusion period, tissue samples from the left ventricular apex ischemic region were analyzed. Tissues were homogenized (100 mg/ml) in 1.15% KCl buffer. Myocardial MDA, SOD, NOS, and NO content were determined per manufacturer protocol.

### In situ cell apoptosis detection

Myocardial sections (5 μm thick) were stained with terminal deoxynucleotidyl transferase-mediated dUTP nick end-labeling (TUNEL) (Roche). After reperfusion, the heart was quickly removed and incubated with 4% paraformaldehyde overnight at room temperature. Heart samples were treated per manufacturer’s protocol. In brief, hearts were fixed with 10% paraformaldehyde and incubated with the TUNEL reaction mixture containing TdT-mediated dUTP nick end labeling. Nuclei were counterstained with 4′,6-diamidino-2-phenylindole (DAPI). Heart samples were visualized on an Olympus FV1000 Laser scanning confocal microscope, and digital images were acquired with IP Lab Imagine Analysis Software (version 3.5, Scanalytics). Apoptotic index was calculated as the percentage of stained, apoptotic cells × 100/total number of nucleated cells.

### Determination of myocardial infarct size

24 hours after reperfusion, infarct size was assessed with Evans Blue (Sigma–Aldrich) and triphenyltetrazolium chloride (TTC; Amresco) staining. At the end of reperfusion period, the coronary artery was immediately retied. 2 mL of 2% Evans Blue solution was administered intravenously to stain the normally perfused region blue. Rat hearts were rapidly excised and frozen at −70°C. Frozen hearts were sliced into 2 mm thick sections parallel to the atrioventricular groove, stained with 1% TTC (pH 7.4) for 15 minutes at 37°C. The viable tissue was stained red by TTC, while the infarct portion not taking up TTC stain remained pale. Infarct area was determined by an image analysis system (Image-Pro plus 3.0; Media Cybernetics). Infarct size was expressed as a percentage of left ventricular volume (%, infarct size/left ventricular).

### H&E staining and immunohistochemistry

After experiment conclusion, left ventricular myocardial ischemic tissue was fixed in neutral formalin, embedded in paraffin, sectioned, stained with hematoxylin and eosin (H&E), and analyzed by light microscopy.

### Reverse transcription and real-time polymerase chain reaction

Hearts were homogenized. Total RNA was extracted by TRIzol (Invitrogen, Shanghai, China) per manufacturer protocol. RNA was treated with RNase-free DNase (Ambion, TX) to eliminate genomic DNA contamination. Total RNA was reverse-transcribed to cDNA by Super-Script II (Invitrogen). Target genes were amplified by standard real-time PCR kit (Sangon Biotech, Co., Ltd, Shanghai, China). RT-PCR was performed in a real-time PCR system (Applied Biosystems, USA) under the following conditions: 95°C denaturation for 2 minutes, followed by 35 cycles of 95°C for 30 seconds and 60°C for 30 seconds. Fold changes in gene expression were calculated after normalizing to β-actin using the formula 2^–Ct^.

### Electron microscopy

Approximately 1 mm^3^ of myocardial tissue was fixed, dipped, and dyed per electron microscope specimen processing requirements. After displacement, the tissue was soaked in Epon 812 epoxy resin and embedded. Simultaneously, 1–2 μm ultrathin slices were prepared. After polymerization, sections were stained with toluidine blue. Coverslips were placed over the samples. Ultrathin sections (ranging 50–70 nm) were prepared from the surfaces of trimmed blocks by an LKBV ultramicrotome (LKB, Sweden). Sections were observed and photographed with a JEM 1010 electron microscope (JEOL, Japan) after aqueous uranium acetate and lead citrate solution staining.

### Western blot analysis

Frozen ventricle samples (n = 6 rats/group) were homogenized in protein lysate buffer (50 mmol/L Tris–HCl, pH = 7.5, 50 mmol/L 2-mercaptoethanol, 5 mmol/L EGTA, 2 mmol/L EDTA, 1% NP-40, 0.1% SDS, 0.5% deoxycholic acid, 10 mmol/L NaF, 1 mmol/L PMSF, 25 mg/mL leupeptin, 2 mg/mL aprotinin), and protein concentrations were determined as previously described [29,30]. For immunoblotting, 5X loading buffer containing 2- mercaptoethanol was added to the protein samples, followed by boiling at 100°C for 10 minutes before loading to 10% SDS-PAGE gel. After SDS-PAGE, proteins were transferred to a PVDF membrane. The membrane was blocked with 5% nonfat milk for 2 hours at room temperature. Primary antibodies were diluted 1:1000 in TBST, added to the membrane, and incubated overnight with agitation at room temperature. After three TBST washings, the membrane was incubated with a 1:2000 dilution of horseradish peroxidase (HRP)-conjugated goat anti-rabbit IgG at room temperature for 60 minutes. After additional TBST washes, signals were evaluated by an enhanced chemiluminescence detection system.

### Statistical analysis

All values are expressed as means ± SEM. Statistical analysis was performed using repeated measures and one-way ANOVA, followed by the Tukey HSD test. P-values are two-sided, and P-values less than 0.05 were considered significant.

## Results

### Streptozocin administration successfully induced a diabetic model

**Figure 2:**
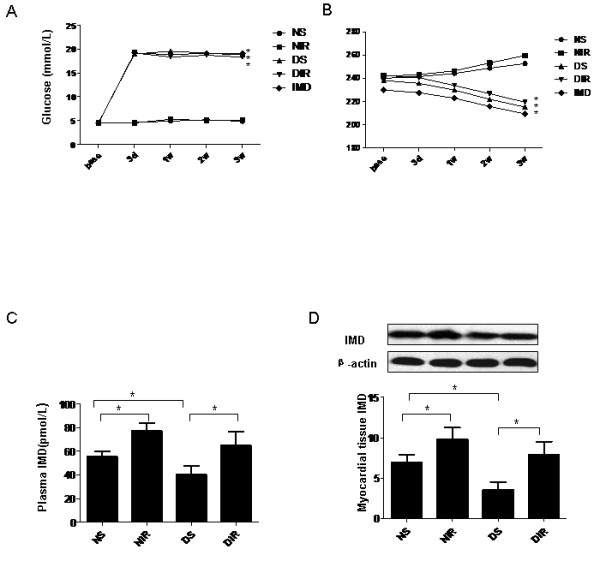
**IMD levels in diabetic rat plasma and myocardial tissue.** (**A**) Animal blood glucose level at designated time points. (**B**) Animal weight at designated time points (**C**) IMD levels in diabetic rat plasma. (**D**) Western blot demonstrating altered IMD expression in myocardial tissue. Image is representative of three separate experiments. Results represent mean ± SEM. *P < 0.05. NS = non-diabetic sham; NIR = non-diabetic I/R treated with vehicle; DS = diabetic sham; DIR = diabetic I/R treated with vehicle; IMD = diabetic I/R treated with IMD.

Weight and blood glucose levels of the rats were recorded at the beginning of the experiment, and 1, 2, and 3 weeks after initial STZ injection. Initial body weight and blood glucose were similar between all groups. 3 weeks after STZ injection, diabetic rats manifested increased blood glucose levels and decreased body weight (P < 0.05, Table 1, Figure 2A, B).

**Table 1:** Animal blood glucose level and weight at designated time points

**Group**	**Glucose (mmol/L)**		**Weight (g)**	
	**baseline**	**After 3 weeks diet**	**baseline**	**After 3 weeks diet**
NS	4.51 ± 0.07	4.82 ± 0.12	239.9 ± 3.52	279.2 ± 4.29*
NIR	4.44 ± 0.09	4.89 ± 0.17	241.7 ± 4.11	271.6 ± 4.03*
DS	4.58 ± 0.08	16.22 ± 2.91*	238.1 ± 5.99	209.1 ± 5.77*
DIR	4.55 ± 0.05	15.84 ± 2.82*	242.5 ± 3.70	213.4 ± 3.61*
IMD	4.53 ± 0.06	16.18 ± 2.91*	229.8 ± 6.02	200.1 ± 5.65*

Abbreviations: *NS*: non-diabetic sham; *NIR*: non-diabetic ischemia/reperfusion + vehicle; *DS*: diabetic sham; DIR: diabetic ischemia/reperfusion + vehicle; *IMD*: diabetic ischemica/reperfusion group + IMD treatment. Results represent mean ± SEM. *P < 0.05 vs. baseline. n = 6-12/group.

### Diabetic animals manifested significantly decreased IMD levels, while MI/R increased IMD levels

IMD levels were significantly decreased in diabetic rats compared to control animals. After MI/R, both plasma and myocardial tissue IMD levels were significantly increased in normal and diabetic rats compared to sham-operated rats, suggesting that MI/R increased IMD levels (P < 0.05, Figure 2C, D).

### IMD attenuated MI/R injury in diabetic animals

**Figure 3:**
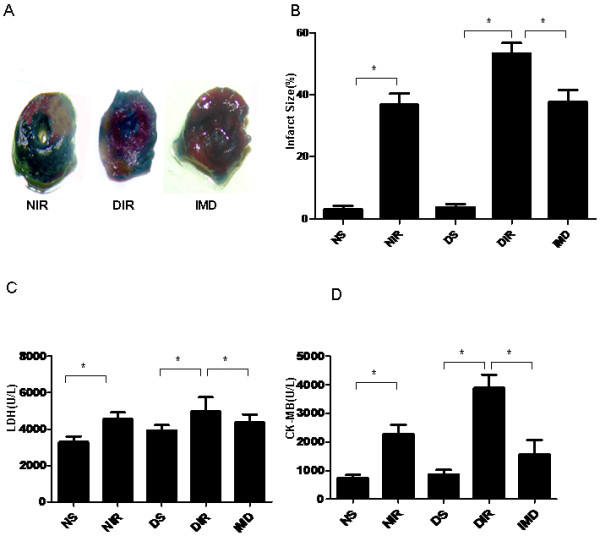
**IMD attenuated MI/R injury in diabetic animals.** (**A**) Representative image of rat hearts stained with TTC. IMD administration decreased (**B**) infarct size (**C**) LDH (**D**) CK-MB after diabetic animals were subjected to MI/R injury. LDH and CK-MB determined after 120 minutes reperfusion. Infarct size determined after 24 hours of reperfusion. n = 5 groups of rats. Results represent mean ± SEM.*P < 0.05. NS = non-diabetic sham; NIR = non-diabetic I/R treated with vehicle; DS = diabetic sham; DIR = diabetic I/R treated with vehicle; IMD = diabetic I/R treated with IMD.

IMD administration significantly reduced infarct size from 40.6% ± 2.5 to 13.2% ± 1.7 in diabetic rats (P < 0.05, Figure 3A, B). IMD treatment decreased serum CK-MB and LDH levels (P < 0.05, Figure 3C, D), and improved left ventricular dysfunction in diabetic rats (augmenting LVDP 82.51 ± 4.53 vs. 60.58 ± 5.47, +dp/dt_max_ 5565 ± 403.0 vs 3877 ± 256.1, and –dp/dt_max_ 4076 ± 280.2 vs. 2830 ± 205.1, all P < 0.05, Table 2), suggesting IMD may protect against MI/R injury in diabetic rats.

**Table 2:** Effects of IMD upon Hemodynamic Parameters Measured After MI

**Parameter (units) / Group**	**NS**	**NIR**	**DS**	**DIR**	**IMD**
LVDP(mmHg)	99.68 ± 2.95	71.81 ± 4.55†	92.91 ± 3.05	60.58 ± 5.47#	82.51 ± 4.53*
+dp/dtmax(mmHg /s)	7584 ± 295.8	5947 ± 354.4†	7012 ± 455.1	3877 ± 256.1#	5565 ± 403.0*
-dp/dtmax(mmHg /s)	5612 ± 148.3	4420 ± 358.9†	5324 ± 213.6	2830 ± 205.1#	4076 ± 280.2*

Abbreviations: *NS*: non-diabetic sham; *NIR*: non-diabetic ischemia/reperfusion + vehicle; *DS*: diabetic sham; *DIR*: diabetic ischemia/reperfusion + vehicle; *IMD*: diabetic ischemica/reperfusion group + IMD treatment. Results represent mean ± SEM. †P < 0.05 vs. NS, #P < 0.05 vs. DS, *P < 0.05 vs. DIR. n = 6-12/group.

### IMD attenuated oxidative stress-induced injury in diabetic rats after MI/R

Malondialdehyde (MDA) is a well-accepted marker of oxidative stress. Post-MI/R MDA levels were significantly increased in diabetic rats, which was markedly reduced by IMD treatment (19.88 ± 1.31 nmol/mg protein, P < 0.05). MI/R increased NOS and NO accumulation, increased expression of reactive oxygen species (ROS)-generating nicotinamide adenine dinucleotide phosphate (NADPH) oxidase (p22phox, p67phox, gp91phox) mRNA, and decreased SOD activity in both diabetic and control rats. IMD administration partially reversed all these outcomes, suggesting IMD blocked MI/R induced oxidative stress in diabetic rats (P < 0.05, Table 3).

**Table 3:** IMD decreases myocardial injury by attenuating oxidative stress

**Marker(units)/Group**	**NS**	**NIR**	**DS**	**DIR**	**IMD**
MDA (nmol/mg prot)	10.61 ± 0.46	17.33 ± 1.60†	15.77 ± 0.95	26.29 ± 0.71#	19.88 ± 1.31*
SOD U/mg prot)	210.6 ± 9.13	158.0 ± 6.58†	201.4 ± 12.52	133.3 ± 9.55#	184.7 ± 14.39*
NOS (U/g prot)	3.48 ± 0.10	2.77 ± 0.22†	2.99 ± 0.11	1.99 ± 0.14#	2.74 ± 0.22*
NO(umol/g prot)	1.58 ± 0.03	0.85 ± 0.02†	1.34 ± 0.02	0.51 ± 0.04#	0.98 ± 0.02*
p22 phox	1.04 ± 0.12	3.03 ± 0.29†	1.77 ± 0.14	4.97 ± 0.46#	2.63 ± 0.14*
p67 phox	0.92 ± 0.08	3.30 ± 0.24†	2.87 ± 0.15	4.37 ± 0.29#	3.20 ± 0.20*
gP91phox	1.07 ± 0.15	3.25 ± 0.29†	2.41 ± 0.23	5.08 ± 0.28#	3.49 ± 0.43*

Abbreviations: *NS*: non-diabetic sham; *NIR*: non-diabetic ischemia/reperfusion + vehicle; *DS*: diabetic sham; *DIR*: diabetic ischemia/reperfusion + vehicle; *IMD*: diabetic ischemica/reperfusion group + IMD treatment. Results represent mean ± SEM. †P < 0.05 vs. NS, #P < 0.05 vs. DS, *P < 0.05 vs. DIR .n = 6-12/group.

### IMD attenuated cardiomyocyte apoptosis in diabetic rats after MI/R

**Figure 4:**
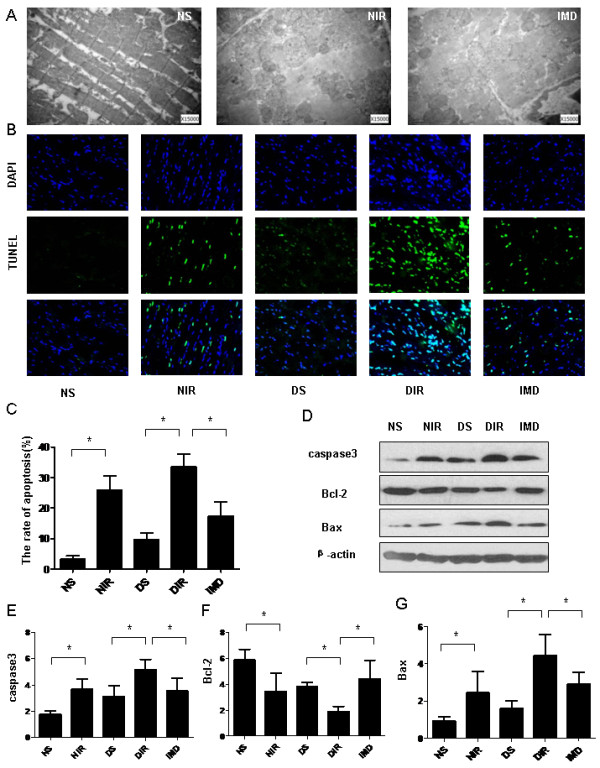
**IMD mitigates myocardial injury by reducing apoptosis.** (**A**) Mitochondrial swelling, visualized with the electron microscope, caused by MI/R of diabetic animals. (**B**) TUNEL assay. Total nuclei labeled by DAPI (blue). Apoptotic nuclei detected by TUNEL staining (green). (**C**) The rate of apoptosis (%) of each group (n = 4-5 sections/group). (**D**) Western blot analysis of activated caspase-3, Bcl-2, and Bax protein in myocardial tissues. Quantitative analysis of (**E**) Caspase-3, (**F**) Bcl-2, and (**G**) Bax Western blots. Results represent mean ± SEM. *P < 0.05. NS = non-diabetic sham; NIR = non-diabetic I/R treated with vehicle; DS = diabetic sham; DIR = diabetic I/R treated with vehicle; IMD = diabetic I/R treated with IMD.

MI/R induced increased cardiomyocyte apoptosis. Electron microscopy revealed IMD administration significantly reduced myocardial ultrastructural mitochondrial damage in the diabetic group. IMD treatment ameliorated cardiomyocyte apoptosis, decreased caspase-3 activity, decreased pro-apoptotic Bax protein expression, and increased anti-apoptotic Bcl-2 protein expression after MI/R in diabetic or normal rats (P < 0.05, Figure 4).

### IMD attenuated I/R-induced inflammation in diabetic rats

**Figure 5:**
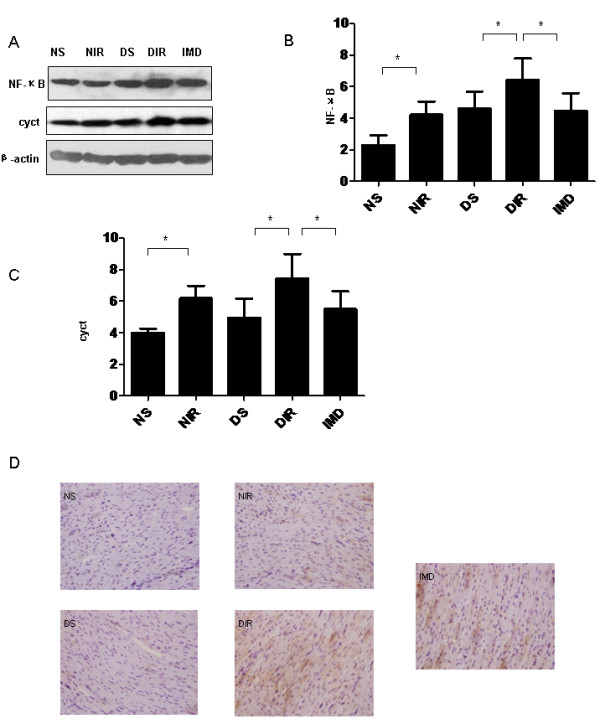
**IMD attenuates myocardial inflammation.** (**A**) Western blot analysis demonstrating IMD treatment inhibited NF-κB and cytochrome C (cytc) protein expression. Quantitative analysis of (**B**) NF-κB (**C**) cytc Western blots. (**D**) Representative histological images for NF-κB expression in formalin-fixed myocardial tissues (400X magnification). Results represent mean ± SEM*P < 0.05. NS = non-diabetic sham; NIR = non-diabetic I/R treated with vehicle; DS = diabetic sham; DIR = diabetic I/R treated with vehicle; IMD = diabetic I/R treated with IMD.

MI/R significantly increased TNF-α, IL-6, and IL-1β levels compared to respective control groups (P < 0.05 Table 4). Importantly, cytokine expression was significantly greater in diabetic mice compared to nondiabetic animals, both in sham and MI/R groups (P < 0.05, Figure 5). IMD reduced TNF-α, IL-6, and IL-1β levels (P < 0.05, Table 4). Nuclear translocation of nuclear transcription factor kappa B (NF-κB) in cardiomyocytes was determined by immunohistochemistry. Western blot analysis determined myocardial NF-κB content and cytochrome C oxidase expression. MI/R increased both myocardial nuclear NF-κB translocation and cytochrome C oxidase expression. Diabetic animals exhibited increased NF-κB expression compared to nondiabetic animals. IMD administration decreased NF-κB protein expression compared to control (P < 0.05 Figure 5).

**Table 4:** IMD decreases myocardial injury by attenuating inflammatory response

**Inflammatory marker(units)/Group**		**NS**	**NIR**	**DS**	**DIR**	**IMD**
Serum	TNF-α(pg/ml)	65.34 ± 1.49	111.7 ± 3.48†	91.26 ± 2.36	197.9 ± 4.87#	111.9 ± 8.85*
	IL-6(pg/ml)	152.2 ± 4.43	287.4 ± 12.44†	183.0 ± 6.23	368.8 ± 12.92#	266.8 ± 20.83*
	IL-1(pg/ml)	29.74 ± 1.62	70.09 ± 3.12†	42.86 ± 2.02	97.23 ± 6.14#	60.59 ± 3.87*
Myocardial	TNF-α(Ct)	2.65 ± 0.14	4.35 ± 0.39†	2.88 ± 0.22	5.22 ± 0.33#	3.64 ± 0.49*
		IL-6(Ct)	1.78 ± 0.81	4.08 ± 0.25†	3.13 ± 0.10	4.47 ± 0.20#	3.18 ± 0.28*
		IL-1(Ct)	1.71 ± 0.17	3.71 ± 0.29†	2.72 ± 0.26	5.25 ± 0.26#	3.97 ± 0.41*

Abbreviations: *NS*: non-diabetic sham; NIR: non-diabetic ischemia/reperfusion + vehicle; *DS*: diabetic sham; *DIR*: diabetic ischemia/reperfusion + vehicle; *IMD*: diabetic ischemica/reperfusion group + IMD treatment. Results represent mean ± SEM. †P < 0.05 vs. NS, #P < 0.05 vs. DS, *P < 0.05 vs. DIR. n = 6-12/group.

## Discussion

To our best knowledge, this is the first study to demonstrate IMD is cardioprotective against MI/R induced injury in diabetic rats. Moreover, we have provided evidence that augmentation of IMD levels after I/R may be a physiologic protective response. Increased plasma levels of intermedin and brain natriuretic peptide are associated more severe coronary stenosis in acute coronary syndrome [31]. In our study, diabetic animals exhibited decreased plasma and myocardial IMD levels.

The response of the hyperglycemic diabetic heart during ischemic injury remains controversial [31-35]. Experimental studies employing ischemia/reperfusion protocols have demonstrated hearts from STZ diabetic rats subjected to a no-flow period of ischemia manifest reduced myocardial infarction (MI) area and recover significantly better ventricular function than nondiabetic hearts, suggesting a possible cardioprotective role of hyperglycemia [36,37]. However, overwhelming epidemiological and clinical data demonstrate the diabetic heart is more sensitive to ischemia-induced injury [38-41]. The metabolic syndrome significantly alters the cardiac gene expression profile, with implications in cardiac pathology development [42].

Recent studies demonstrate STZ-induced diabetes mellitus significantly exacerbates MIR injury, blunting the protective effect of various therapeutic agents [43,44]. IMD has been shown to protect against MI/R-induced injury [11,17]. Myocardial oxidative stress contributes importantly to diabetic pathophysiology. Hyperglycemia enhances oxidative stress, and reduces antioxidant defenses [45]. MDA is an unsaturated fatty acid in free radical and lipid peroxidation metabolites. An indirect marker of cellular damage degree, MDA content reflects the extent of systemic lipid peroxidation. The antioxidant SOD protects cells by reducing free radical-induced injury. SOD levels reflect the body’s capacity to scavenge oxygen free radicals. In the current study, myocardial SOD activity was attenuated in the diabetic animal group, which was further decreased by MI/R. In combination with increased MDA content observed in the diabetic MI/R group, our data suggests hyperglycemia-enhanced oxidative stress may exacerbate MI/R injury.

In hypertrophied cardiomyocytes, intermedin expression is augmented [46]. During oxidative stress, reactive oxygen species (ROS) damage biological molecules such as DNA and proteins. Notably, NADPH produces superoxide anion O^2-^, generating cell-damaging H_2_O_2,_ which mediates cardiomyocyte apoptosis [47,48]. Reduced nicotinamide adenine dinucleotide phosphate (NADPH) oxidase complexes, normally distributed in ventricular muscle and vascular smooth muscle cells, increases in vivo ROS [49-52]. Diabetic cardiomyopathy is characterized by increased myocardial NADPH oxidase (specifically isoforms p22phox, p67phox, and gp91phox) expression, leading to increased myocardial ROS generation and lipid peroxidation [53-56]. We demonstrate in the current study that IMD decreased expression of p22, p67 and gp91.

We provide evidence that IMD preserves and regulates NO. In our study, myocardial NOS activity and NO were significantly decreased in both nondiabetic and diabetic animals after MI/R. Previously, intermedin has been shown to exert negative inotropic effects in Langendorff-perfused rat hearts, an effect blocked by inhibition of nitric oxide synthesis [57]. Another study demonstrated IMD increased endothelial nitric oxide synthase (eNOS) phosphorylation nearly three-fold at Ser (1177), significantly enhancing eNOS activity [58]. In the current study, IMD administration preserved myocardial NOS activity and cardiac NO levels, suggesting IMD regulates both myocardial NOS activity and NO production.

The relationship between diabetic cardiomyopathy and cellular apoptosis is well known [45,59-61]. Previous studies demonstrate IMD administration decreases MI/R-induced cardiomyocyte apoptosis [11,17], and myocardial injury may be exacerbated by downregulated IMD during early reperfusion [62]. We demonstrate MI/R increased caspase-3 activity and Bax protein expression in diabetic animals compared to control, which was attenuated by IMD administration. These data reinforce previous reports that diabetic rats manifest exacerbated injury after MI/R compared to non-diabetic rats [63,64]. Inflammatory cytokines mediate critical pathologic effects during MI/R [65,66]. The diabetic condition, accepted now as a state of low-level, chronic inflammation, predisposes to significantly enhanced I/R-induced myocardial inflammation [67]. In our current study, IMD administration decreased diabetes-induced myocardial NF-κB activation, cytochrome C oxidase expression, and serum/myocardial TNF-α, IL-1, and IL-6 expression. Further investigations confirming whether IMD attenuates diabetic MI/R injury via inflammatory signaling mitigation are warranted.

## Conclusions

Myocardial ischemia-reperfusion injury is multifactorial. The current study demonstrated IMD administration reduces hyperglycemia-exacerbated MI/R injury via reduction of oxidative stress, apoptosis, and inflammation in a diabetic rat model. We provide evidence suggesting IMD is a cardioprotective molecule in diabetic animals after MI/R. Further studies investigating the specific mechanisms by which IMD exerts its cardioprotective effects are ongoing. IMD may be a promising novel therapeutic target against diabetic ischemic heart disease.

## Competing interests

The authors declare that they have no competing interest.

## Authors’ contributions

CSX, YFB, and HL conceived the study and participated in its design. HL and WBL drafted the manuscript. HL performed the statistical analyses. HL, NZ, JG, and CW interpreted the data. All authors have read and approved the final manuscript.

## References

[B1] Gross ER Hsu AK Gross GJ Diabetes abolishes morphine-induced cardioprotection via multiple pathways upstream of glycogen synthase kinase-3beta Diab 2007 56 1 127 136 10.2337/db06-0907 17192474

[B2] Ametov AS P'Ianykh OP Aslandziia EN Acute coronary syndrome in patients with type 2 diabetes mellitus Ter Arkh 2011 83 9 66 70 22145391

[B3] Kappert K Bohm M Schmieder R Schumacher H Teo K Yusuf S Sleight P Unger T Impact of sex on cardiovascular outcome in patients at high cardiovascular risk: analysis of the Telmisartan Randomized Assessment Study in ACE-Intolerant Subjects With Cardiovascular Disease (TRANSCEND) and the Ongoing Telmisartan Alone and in Combination With Ramipril Global End Point Trial (ONTARGET) Circ 2012 126 8 934 941 10.1161/CIRCULATIONAHA.111.086660 22829023

[B4] Tanaka K Kehl F Gu W Krolikowski JG Pagel PS Warltier DC Kersten JR Isoflurane-induced preconditioning is attenuated by diabetes Am J Physiol Heart Circ Physiol 2002 282 6 H2018 2023 12003806 10.1152/ajpheart.01130.2001

[B5] Hotta H Miura T Miki T Togashi N Maeda T Kim SJ Tanno M Yano T Kuno A Itoh T Angiotensin II type 1 receptor-mediated upregulation of calcineurin activity underlies impairment of cardioprotective signaling in diabetic hearts Circ Res 2010 106 1 129 132 10.1161/CIRCRESAHA.109.205385 19910577

[B6] Winer N Sowers JR Epidemiology of diabetes J Clin Pharmacol 2004 44 4 397 405 10.1177/0091270004263017 15051748

[B7] Kannel WB McGee DL Diabetes and cardiovascular disease. The Framingham study JAMA 1979 241 19 2035 2038 10.1001/jama.1979.03290450033020 430798

[B8] Danaei G Friedman AB Oza S Murray CJ Ezzati M Diabetes prevalence and diagnosis in US states: analysis of health surveys Popul health metrics 2009 7 16 10.1186/1478-7954-7-16 PMC2764564 19781056

[B9] Potier L Waeckel L Vincent MP Chollet C Gobeil F Marre M Bruneval P Richer C Roussel R Alhenc-Gelas F Selective kinin receptor agonists as cardioprotective agents in myocardial ischemia and diabetes J Pharmacol Exp Ther 2013 [Epub ahead of print] 10.1124/jpet.113.203927 23591995

[B10] Roh J Chang CL Bhalla A Klein C Hsu SY Intermedin is a calcitonin/calcitonin gene-related peptide family peptide acting through the calcitonin receptor-like receptor/receptor activity-modifying protein receptor complexes J Biol Chem 2004 279 8 7264 7274 14615490 10.1074/jbc.M305332200

[B11] Yang JH Qi YF Jia YX Pan CS Zhao J Yang J Chang JK Tang CS Protective effects of intermedin/adrenomedullin2 on ischemia/reperfusion injury in isolated rat hearts Pept 2005 26 3 501 507 10.1016/j.peptides.2004.10.025 15652657

[B12] Bell D Campbell M Ferguson M Sayers L Donaghy L O'Regan A Jewhurst V Harbinson M AM(1)-receptor-dependent protection by intermedin of human vascular and cardiac non-vascular cells from ischaemia-reperfusion injury J Physiol 2012 590 Pt 5 1181 1197 22183724 10.1113/jphysiol.2011.221895 PMC3381824

[B13] Hirose T Totsune K Mori N Morimoto R Hashimoto M Nakashige Y Metoki H Asayama K Kikuya M Ohkubo T Increased expression of adrenomedullin 2/intermedin in rat hearts with congestive heart failure Eur J Heart Fail 2008 10 9 840 849 10.1016/j.ejheart.2008.06.020 18692436

[B14] Dong F Taylor MM Samson WK Ren J Intermedin (adrenomedullin-2) enhances cardiac contractile function via a protein kinase C- and protein kinase A-dependent pathway in murine ventricular myocytes J Appl Physiol (Bethesda, Md : 1985) 2006 101 3 778 784 10.1152/japplphysiol.01631.2005 16763098

[B15] Yang JH Cai Y Duan XH Ma CG Wang X Tang CS Qi YF Intermedin 1–53 inhibits rat cardiac fibroblast activation induced by angiotensin II Regul Pept 2009 158 1–3 19 25 19523990 10.1016/j.regpep.2009.05.012

[B16] Pan CS Yang JH Cai DY Zhao J Gerns H Yang J Chang JK Tang CS Qi YF Cardiovascular effects of newly discovered peptide intermedin/adrenomedullin 2 Pept 2005 26 9 1640 1646 10.1016/j.peptides.2005.02.013 16112404

[B17] Du QX Yue W Wang YY [Effect and mechanism of intermedin in acute rat cardiac ischemic injury] Fa yi xue za zhi 2011 27 3 164 168 21899003

[B18] Teng X Song J Zhang G Cai Y Yuan F Du J Tang C Qi Y Inhibition of endoplasmic reticulum stress by intermedin(1–53) protects against myocardial injury through a PI3 kinase-Akt signaling pathway J Mol Med (Berlin, Germany) 2011 89 1205 1195 10.1007/s00109-011-0808-5 21909975

[B19] Song JQ Teng X Cai Y Tang CS Qi YF Activation of Akt/GSK-3beta signaling pathway is involved in intermedin(1–53) protection against myocardial apoptosis induced by ischemia/reperfusion Apoptosis 2009 14 11 1299 1307 10.1007/s10495-009-0398-7 19757065

[B20] He RX Gu CL Shen F Zhang XM Changes in expression of adrenomedullin in the myocardium of streptozotocin-induced diabetic rats Chin Med J 2007 120 3 187 191 17355819

[B21] Soetikno V Watanabe K Sari FR Harima M Thandavarayan RA Veeraveedu PT Arozal W Sukumaran V Lakshmanan AP Arumugam S Curcumin attenuates diabetic nephropathy by inhibiting PKC-alpha and PKC-beta1 activity in streptozotocin-induced type I diabetic rats Mol Nutr Food Res 2011 55 11 1655 1665 10.1002/mnfr.201100080 22045654

[B22] Kromann H Christy M Lernmark A Nedergaard M Nerup J The low dose streptozotocin murine model of type 1 (insulin-dependent) diabetes mellitus: studies in vivo and in vitro of the modulating effect of sex hormones Diabetologia 1982 22 3 194 198 6210590 10.1007/BF00283752

[B23] Fang J Chen L Wu L Li W Intra-cardiac remote ischemic post-conditioning attenuates ischemia-reperfusion injury in rats Scand Cardiovas J: SCJ 2009 43 6 386 394 10.1080/14017430902866681 19353379

[B24] Gao Y Yao X Zhang Y Li W Kang K Sun L Sun X The protective role of hydrogen sulfide in myocardial ischemia-reperfusion-induced injury in diabetic rats Int J Cardiol 2011 152 2 177 183 10.1016/j.ijcard.2010.07.012 21316771

[B25] Zhang HY Jiang W Liu JY Li Y Chen CL Xin HB Huang DJ Intermedin is upregulated and has protective roles in a mouse ischemia/reperfusion model Hypertens Res 2009 32 10 861 868 10.1038/hr.2009.120 19680258

[B26] Zhang L Ma J Liu H Protective effect of ischemic postconditioning against ischemia reperfusion-induced myocardium oxidative injury in IR rats Mol 2012 17 4 3805 3817 10.3390/molecules17043805 PMC6268873 22453931

[B27] Cao J Vecoli C Neglia D Tavazzi B Lazzarino G Novelli M Masiello P Wang YT Puri N Paolocci N Cobalt-Protoporphyrin Improves Heart Function by Blunting Oxidative Stress and Restoring NO Synthase Equilibrium in an Animal Model of Experimental Diabetes Front Physiol 2012 3 160 22675305 10.3389/fphys.2012.00160 PMC3366474

[B28] Okazaki T Otani H Shimazu T Yoshioka K Fujita M Iwasaka T Ascorbic acid and N-acetyl cysteine prevent uncoupling of nitric oxide synthase and increase tolerance to ischemia/reperfusion injury in diabetic rat heart Free Radic Res 2011 45 10 1173 1183 10.3109/10715762.2011.605361 21756052

[B29] Rajesh M Mukhopadhyay P Batkai S Patel V Saito K Matsumoto S Kashiwaya Y Horvath B Mukhopadhyay B Becker L Cannabidiol attenuates cardiac dysfunction, oxidative stress, fibrosis, and inflammatory and cell death signaling pathways in diabetic cardiomyopathy J Am Coll Cardiol 2010 56 25 2115 2125 10.1016/j.jacc.2010.07.033 21144973 PMC3026637

[B30] De Waard MC van der Velden J Boontje NM Dekkers DH Van Haperen R Kuster DW Lamers JM De Crom R Duncker DJ Detrimental effect of combined exercise training and eNOS overexpression on cardiac function after myocardial infarction Am J Physiol Heart Circ Physiol 2009 296 5 H1513 1523 10.1152/ajpheart.00485.2008 19286956

[B31] Qin YW Teng X He JQ Du J Tang CS Qi YF Increased plasma levels of intermedin and brain natriuretic peptide associated with severity of coronary stenosis in acute coronary syndrome Pept 2013 42 84 88 10.1016/j.peptides.2013.01.011 23391507

[B32] Feuvray D Lopaschuk GD Controversies on the sensitivity of the diabetic heart to ischemic injury: the sensitivity of the diabetic heart to ischemic injury is decreased Cardiovasc Res 1997 34 1 113 120 10.1016/S0008-6363(97)00037-0 9217880

[B33] Ravingerova T Neckar J Kolar F Stetka R Volkovova K Ziegelhoffer A Styk J Ventricular arrhythmias following coronary artery occlusion in rats: is the diabetic heart less or more sensitive to ischaemia? Basic Res Cardiol 2001 96 2 160 168 10.1007/s003950170066 11327334

[B34] Backlund T Palojoki E Saraste A Eriksson A Finckenberg P Kyto V Lakkisto P Mervaala E Voipio-Pulkki LM Laine M Sustained cardiomyocyte apoptosis and left ventricular remodelling after myocardial infarction in experimental diabetes Diabetologia 2004 47 2 325 330 10.1007/s00125-003-1311-5 14722653

[B35] Miki T Itoh T Sunaga D Miura T Effects of diabetes on myocardial infarct size and cardioprotection by preconditioning and postconditioning Cardiovasc Diabetol 2012 11 67 10.1186/1475-2840-11-67 22694800 PMC3461466

[B36] Rodrigues B Rosa KT Medeiros A Schaan BD Brum PC De Angelis K Irigoyen MC Hyperglycemia can delay left ventricular dysfunction but not autonomic damage after myocardial infarction in rodents Cardiovasc Diabetol 2011 10 26 10.1186/1475-2840-10-26 21470409 PMC3084163

[B37] Chu LM Osipov RM Robich MP Feng J Oyamada S Bianchi C Sellke FW Is hyperglycemia bad for the heart during acute ischemia? J Thorac Cardiovasc Surg 2010 140 6 1345 1352 10.1016/j.jtcvs.2010.05.009 20542299 PMC2949689

[B38] Lekli I Szabo G Juhasz B Das S Das M Varga E Szendrei L Gesztelyi R Varadi J Bak I Protective mechanisms of resveratrol against ischemia-reperfusion-induced damage in hearts obtained from Zucker obese rats: the role of GLUT-4 and endothelin Am J Physiol Heart Circ Physiol 2008 294 2 H859 866 10.1152/ajpheart.01048.2007 18065527

[B39] Chu LM Osipov RM Robich MP Feng J Sheller MR Sellke FW Effect of thrombin fragment (TP508) on myocardial ischemia reperfusion injury in a model of type 1 diabetes mellitus Circ 2010 122 11 Suppl S162 169 10.1161/CIRCULATIONAHA.109.928374 PMC2943853 20837908

[B40] Marso SP Miller T Rutherford BD Gibbons RJ Qureshi M Kalynych A Turco M Schultheiss HP Mehran R Krucoff MW Comparison of myocardial reperfusion in patients undergoing percutaneous coronary intervention in ST-segment elevation acute myocardial infarction with versus without diabetes mellitus (from the EMERALD Trial) Am J Cardiol 2007 100 2 206 210 10.1016/j.amjcard.2007.02.080 17631071

[B41] Alegria JR Miller TD Gibbons RJ Yi QL Yusuf S Infarct size, ejection fraction, and mortality in diabetic patients with acute myocardial infarction treated with thrombolytic therapy Am Heart J 2007 154 4 743 750 10.1016/j.ahj.2007.06.020 17893003

[B42] Sarkozy M Zvara A Gyemant N Fekete V Kocsis GF Pipis J Szucs G Csonka C Puskas LG Ferdinandy P Metabolic syndrome influences cardiac gene expression pattern at the transcript level in male ZDF rats Cardiovasc Diabetol 2013 12 16 10.1186/1475-2840-12-16 23320804 PMC3599923

[B43] Hu ZC Chen YD Ren YH Methylprednisolone improves microcirculation in streptozotocin-induced diabetic rats after myocardial ischemia/reperfusion Chin Med J 2011 124 6 923 929 21518604

[B44] Wu Y Xia ZY Dou J Zhang L Xu JJ Zhao B Lei S Liu HM Protective effect of ginsenoside Rb1 against myocardial ischemia/reperfusion injury in streptozotocin-induced diabetic rats Mol Biol Rep 2011 38 7 4327 4335 10.1007/s11033-010-0558-4 21113666

[B45] Kain V Kumar S Sitasawad SL Azelnidipine prevents cardiac dysfunction in streptozotocin-diabetic rats by reducing intracellular calcium accumulation, oxidative stress and apoptosis Cardiovasc Diabetol 2011 10 97 10.1186/1475-2840-10-97 22054019 PMC3234183

[B46] Bell D Zhao Y McCoy FP Devine A McDermott BJ Expression of the counter-regulatory peptide intermedin is augmented in the presence of oxidative stress in hypertrophied cardiomyocytes Cell Physiol Biochem 2008 21 5–6 409 420 18453748 10.1159/000129633

[B47] Cifuentes ME Pagano PJ Targeting reactive oxygen species in hypertension Curr Opin Nephrol Hypertens 2006 15 2 179 186 10.1097/01.mnh.0000214776.19233.68 16481886

[B48] Ibrahim HM El-Elaimy IA Saad Eldien HM Badr BM Rabah DM Badr G Blocking type I interferon signaling rescues lymphocytes from oxidative stress, exhaustion, and apoptosis in a streptozotocin-induced mouse model of type I diabetes Oxid Med Cell Longev 2013 2013 148725 23533683 10.1155/2013/148725 PMC3606800

[B49] Isabelle M Vergeade A Moritz F Dautreaux B Henry JP Lallemand F Richard V Mulder P Thuillez C Monteil C NADPH oxidase inhibition prevents cocaine-induced up-regulation of xanthine oxidoreductase and cardiac dysfunction J Mol Cell Cardiol 2007 42 2 326 332 10.1016/j.yjmcc.2006.11.011 17217956

[B50] Heymes C Bendall JK Ratajczak P Cave AC Samuel JL Hasenfuss G Shah AM Increased myocardial NADPH oxidase activity in human heart failure J Am Coll Cardiol 2003 41 12 2164 2171 10.1016/S0735-1097(03)00471-6 12821241

[B51] Li JM Gall NP Grieve DJ Chen M Shah AM Activation of NADPH oxidase during progression of cardiac hypertrophy to failure Hypertens 2002 40 4 477 484 10.1161/01.HYP.0000032031.30374.32 12364350

[B52] Xiao L Pimentel DR Wang J Singh K Colucci WS Sawyer DB Role of reactive oxygen species and NAD(P)H oxidase in alpha(1)-adrenoceptor signaling in adult rat cardiac myocytes Am J Physiol Cell Physiol 2002 282 4 C926 934 10.1152/ajpcell.00254.2001 11880281

[B53] Soetikno V Sari FR Sukumaran V Lakshmanan AP Mito S Harima M Thandavarayan RA Suzuki K Nagata M Takagi R Curcumin prevents diabetic cardiomyopathy in streptozotocin-induced diabetic rats: possible involvement of PKC-MAPK signaling pathway Eur J Pharm Sci 2012 47 3 604 614 10.1016/j.ejps.2012.04.018 22564708

[B54] Yoshida K Kobayashi N Ohno T Fukushima H Matsuoka H Cardioprotective effect of angiotensin II type 1 receptor antagonist associated with bradykinin-endothelial nitric oxide synthase and oxidative stress in Dahl salt-sensitive hypertensive rats J Hypertens 2007 25 8 1633 1642 10.1097/HJH.0b013e32814db89f 17620960

[B55] Becher PM Lindner D Frohlich M Savvatis K Westermann D Tschope C Assessment of cardiac inflammation and remodeling during the development of streptozotocin-induced diabetic cardiomyopathy in vivo: A time course analysis Int J Mol Med 2013 10.3892/ijmm.2013.1368 23652584

[B56] El-Benna J Dang PM Perianin A Towards specific NADPH oxidase inhibition by small synthetic peptides Cell Mol Life Sci 2012 69 14 2307 2314 10.1007/s00018-012-1008-3 22562604 PMC11114506

[B57] Munzel G Schlier A Schreckenberg R Abdallah Y Schluter KD Rat intermedin1-47 does not improve functional recovery in postischemic hearts Naunyn Schmiedebergs Arch Pharmacol 2011 384 6 535 542 10.1007/s00210-011-0680-4 21881857

[B58] Pires AL Pinho M Alves BS Pinho S Sena C Seica RM Leite-Moreira AF Reverse myocardial effects of intermedin in pressure-overloaded hearts: role of endothelial nitric oxide synthase activity J Physiol 2013 591 Pt 3 677 687 23165766 10.1113/jphysiol.2012.240812 PMC3577549

[B59] Ou HC Tzang BS Chang MH Liu CT Liu HW Lii CK Bau DT Chao PM Kuo WW Cardiac contractile dysfunction and apoptosis in streptozotocin-induced diabetic rats are ameliorated by garlic oil supplementation J Agric Food Chem 2010 58 19 10347 10355 10.1021/jf101606s 20836552

[B60] Li CJ Zhang QM Li MZ Zhang JY Yu P Yu DM Attenuation of myocardial apoptosis by alpha-lipoic acid through suppression of mitochondrial oxidative stress to reduce diabetic cardiomyopathy Chin Med J 2009 122 21 2580 2586 19951573

[B61] Shirpoor A Salami S Khadem-Ansari MH Ilkhanizadeh B Pakdel FG Khademvatani K Cardioprotective effect of vitamin E: rescues of diabetes-induced cardiac malfunction, oxidative stress, and apoptosis in rat J Diabetes Complications 2009 23 5 310 316 10.1016/j.jdiacomp.2008.02.009 18394933

[B62] Teng X Bian Y Cai Y Duan X Yuan F Du J Wu W Wang X Tang C Qi Y Downregulation of endogenous intermedin augmented myocardial injury in rats with ischemia/reperfusion Horm Metab Res 2013 45 3 206 212 23018870 10.1055/s-0032-1327572

[B63] Xiao CY Chen M Zsengeller Z Szabo C Poly(ADP-ribose) polymerase contributes to the development of myocardial infarction in diabetic rats and regulates the nuclear translocation of apoptosis-inducing factor J Pharmacol Exp Ther 2004 310 2 498 504 10.1124/jpet.104.066803 15054118

[B64] Di Filippo C Marfella R Cuzzocrea S Piegari E Petronella P Giugliano D Rossi F D'Amico M Hyperglycemia in streptozotocin-induced diabetic rat increases infarct size associated with low levels of myocardial HO-1 during ischemia/reperfusion Diabetes 2005 54 3 803 810 10.2337/diabetes.54.3.803 15734859

[B65] Dandona P Aljada A Chaudhuri A Mohanty P Garg R Metabolic syndrome: a comprehensive perspective based on interactions between obesity, diabetes, and inflammation Circ 2005 111 11 1448 1454 10.1161/01.CIR.0000158483.13093.9D 15781756

[B66] Turer AT Hill JA Pathogenesis of myocardial ischemia-reperfusion injury and rationale for therapy Am J Cardiol 2010 106 3 360 368 10.1016/j.amjcard.2010.03.032 20643246 PMC2957093

[B67] Wang Y Schmeichel AM Iida H Schmelzer JD Low PA Enhanced inflammatory response via activation of NF-kappaB in acute experimental diabetic neuropathy subjected to ischemia-reperfusion injury J Neurol Sci 2006 247 1 47 52 10.1016/j.jns.2006.03.011 16631800

